# Healthcare resource utilisation and direct medical cost for individuals with 5q spinal muscular atrophy in Sweden

**DOI:** 10.1007/s10198-024-01678-y

**Published:** 2024-04-20

**Authors:** Thomas Sejersen, Sophie Graham, Anne-Berit Ekström, Anna-Karin Kroksmark, Marta Kwiatkowska, Michael L. Ganz, Nahila Justo, Karl Gertow, Alex Simpson

**Affiliations:** 1 Department of Child Neurology, Astrid Lindgren Children’s Hospital, Karolinska University Hospital, Stockholm, Sweden; 2 Department of Women’s and Children’s Health, Karolinska Institute, Stockholm, Sweden; 3 Center for Neuromusculoskeletal Restorative Medicine, Hong Kong Science Park, Shatin, New Territories, Hong Kong; 4 Evidera Ltd, London, UK; 5 Pediatric Rehabilitation Center, Queen Silvia Children’s Hospital, Sahlgrenska University Hospital, Gothenburg, Sweden; 6 Department of Pediatrics, Sahlgrenska Academy, University of Gothenburg, Gothenburg, Sweden; 7 Queen Silvia Children’s Hospital, Sahlgrenska University Hospital, Gothenburg, Sweden; 8 Evidera Inc, Waltham, USA; 9 Evidera-PPD, Stockholm, Sweden; 10 NVS Department, Karolinska Institute, Stockholm, Sweden; 11 Roche AB, Solna, Sweden; 12 F. Hoffmann-La Roche Ltd, Basel, Switzerland

**Keywords:** Cohort study, Direct medical costs, Healthcare resource use, Spinal muscular atrophy, Sweden, Registries, I11

## Abstract

**Background:**

Spinal muscular atrophy (SMA) is a rare, progressive, neuromuscular disorder. Recent advances in treatment require an updated assessment of burden to inform reimbursement decisions.

**Objectives:**

To quantify healthcare resource utilisation (HCRU) and cost of care for patients with SMA.

**Methods:**

Cohort study of patients with SMA identified in the Swedish National Patient Registry (2007–2018), matched to a reference cohort grouped into four SMA types (1, 2, 3, unspecified adult onset [UAO]). HCRU included inpatient admissions, outpatient visits, procedures, and dispensed medications. Direct medical costs were estimated by multiplying HCRU by respective unit costs. Average annual HCRU and medical costs were modelled for SMA versus reference cohorts to estimate differences attributable to the disease (i.e., average treatment effect estimand). The trajectory of direct costs over time were assessed using synthetic cohorts.

**Results:**

We identified 290 SMA patients. Annualised HCRU was higher in SMA patients compared with reference cohorts. Highest risk ratios were observed for inpatient overnight stays for type 1 (risk ratio [RR]: 29.2; 95% confidence interval [CI]: 16.0, 53.5) and type 2 (RR: 23.3; 95% CI: 16.4,33.1). Mean annual direct medical costs per patient for each year since first diagnosis were greatest for type 1 (€114,185 and SMA-attributable: €113,380), type 2 (€61,876 and SMA-attributable: €61,237), type 3 (€45,518 and SMA-attributable: €44,556), and UAO (€4046 and SMA-attributable: €2098). Costs were greatest in the 2–3 years after the first diagnosis for all types.

**Discussion and conclusion:**

The economic burden attributable to SMA is significant. Further research is needed to understand the burden in other European countries and the impact of new treatments.

**Supplementary Information:**

The online version contains supplementary material available at 10.1007/s10198-024-01678-y.

## Introduction

5q spinal muscular atrophy (SMA) is a rare, progressive, neuromuscular disorder that is characterised by skeletal muscle weakness, hypotonia, respiratory deficiency, and a decline in motor function [1–3]. Type 1 is the most severe form of SMA because symptoms usually present at less than six months of age and patients never sit unsupported [1, 2, 4]. Symptomatic individuals with SMA type 1 typically do not experience motor milestones or motor function gains, and without respiratory intervention their life expectancy is generally less than two years [1]. SMA type 1 is the most frequently occurring subtype (> 50% of cases), but it is the least prevalent because of its high mortality rate [1, 5, 6]. SMA type 2 symptoms manifest in the first 6–18 months of life [7]; individuals at some point achieve independent sitting, but never walk unaided, and are expected to live into early adulthood [4, 7]. SMA type 3 usually presents after 18 months of age; affected individuals achieve the ability to walk independently, and their life expectancy is that of the general population [7]. SMA type 4 is the rarest form of the disorder, associated with the lowest morbidity, with symptom onset typically after 21 years of age [7]. Similar to SMA type 3, life expectancy in type 4 is the same as in the general population [4, 7, 8].

Until 2017, therapeutic options for patients with SMA were limited to symptom management [9]. In 2017, the European Medicines Agency approved the first disease-modifying therapy, nusinersen, for use in patients with SMA [10]. Onasemnogene abeparvovec was later approved in 2020 [11] and risdiplam in 2021 [12, 13]. These therapies slow the progression of SMA, especially if administered early in the course of the disorder [13].

Given the debilitating effects of SMA, managing it is resource intensive and the healthcare and economic burden are dependent on the type and severity of SMA [14–18]. A systematic literature review quantified the direct cost of SMA globally and summarised that the annual costs, excluding drug costs, of type 1 SMA ranged from €67,934 to €177,811 [at 2021 prices] [16, 19]. Two other recent systematic literature reviews [17, 18] and other studies conducted since [20–22] have also summarised the high direct medical costs for these patients, however, evidence on the cost of illness in patients with SMA is relatively scarce globally, with very few studies conducted in Europe. These studies also mostly include data from surveys or interviews, which are limited in sample size and generelisability [23].

With the recent advancements in treatments for patients with SMA, updated estimates on healthcare resource utilisation (HCRU) and direct medical costs stratified by SMA subtypes are required to determine the cost-effectiveness of new treatments, which could eventually inform reimbursement decision-making and value-based allocation of resources. Therefore, the aim of this study was (1) to quantify the HCRU and direct medical costs by SMA type and compare them with those of a matched-reference cohort from the general population in Sweden, and (2) to assess the trajectory of HCRU and direct medical costs from the date of symptom onset.

## Methods

### Study design and data sources

This was a population-based matched-reference cohort study of patients with SMA, identified in the Swedish National Patient Registry (NPR) from January 1, 1987 to December 31, 2018. Data were extracted from six Swedish registers: the NPR, Prescribed Drug Register (SPDR), Register of Interventions in Municipal Health Care (HSL), Cause of Death Register (CDR), the Total Population Register (TPR), and the Swedish National Registry for Neuromuscular Disorders (NMiS). The NPR contains nationwide data on hospital stays since 1987 and specialist care for outpatients since 2001. The SPDR has information on drug utilisation and expenditures for prescribed drugs for the entire Swedish population since 2005. It contains only medications dispensed in community pharmacies and not medications administered in the hospitals. The HSL includes information on primary care services provided by the municipality e.g., a nurse or physician home visit provided each month of the year since 2006. The CDR contains death dates and underlying and contributory causes of death since 1965. The TPR has demographic characteristics, immigration and emigration information, and relationships inferred from the Multi-Generation Registry, since 1968. NMiS has collected information since 2011 on confirmed SMA diagnoses, including SMA subtype (1–4) and functional level. Prior to data delivery, the registry holders linked the patient data in these registries using the personal identification numbers corresponding to each individual [24].

### Study population

Data to assess the outcomes of interest were collected from January 1, 2007 to December 31, 2019. To maximise sample size and statistical power, we included both patients diagnosed with SMA before January 1, 2007 (i.e., prevalent SMA cases) and cases identified after January 1, 2007 (i.e., incident SMA cases) which also allowed for the assessment of the disorder over the course of an individuals’ lifetime. Incident SMA cases were identified as having a first-ever SMA-related inpatient or outpatient care event (International Classification of Diseases, 10th Revision, Swedish Version G12.0 or G12.1) in the NPR from January 1, 2007 until December 31, 2018. Prevalent patients were identified as those having a first-ever SMA-related inpatient or outpatient care event with a diagnosis coded as ICD-10-SE G12.0 or G12.1 (or ICD-9-SE 335A, 335B before 1997) in any position in the NPR, between January 1, 1987 and December 31, 2006 and who were alive and had not emigrated as of January 1, 2007. To minimise disease misclassification, both incident and prevalent cases were excluded if they ever had a confirmed diagnosis (two outpatient or one inpatient) of any neuromotor disease other than SMA (ICD-10-SE G12.2, G12.8, or G12.9).

The index date was the date of first diagnosis for incident cases and their corresponding matches and January 1, 2007 for prevalent cases and their matches. Date of first ever SMA diagnosis identified in the data would represent first SMA symptom onset, although in practice this date represented first interaction with specialist or inpatient care for which an SMA diagnosis was recorded. Mutually exclusive SMA types were defined based on the subtype recorded in the NMiS. For patients not included in the NMiS, subtype classification was defined as follows, based on the current literature [25, 26]:

- Type 1 SMA: Patients whose disease was consistently recorded as G12.0 in NPR (i.e., no other ICD-10 code recorded during all follow-up), provided that the first record of an ICD-10-SE G12 or ICD-9-SE 335 diagnosis appeared in the data on or before 12 months of age.
- Type 2 SMA: Patients whose disease was consistently recorded as G12.1A in the NPR, provided that the first record of an ICD-10-SE G12 or ICD-9-SE 335 appeared in the dataset on or before they were 36 months of age.
- Type 3 SMA: Patients whose disease was consistently recorded as G12.1B in the NPR (no age limit).
- Unspecified adult onset (UAO) SMA: Patients with a first diagnosis of G12.1 on or after 18 years of age with at least two records of SMA diagnosis from neurology departments with no subtype assigned.

The patients were matched 1:4, based on sex, age, and county of residence at index, to a reference cohort without SMA. Subjects in the matched reference cohort were selected from the Total Population Register (TPR), which includes all residents in Sweden. They were selected randomly and without replacement. All patients were followed until death, emigration, or the end of the study period, whichever occurred first. The individuals in the reference cohort were censored at the time of death of the case to which they were matched.

### Study measures

#### Variables at index

- Demographic characteristics evaluated at index date: Age and sex.
- Disease presentation evaluated at index: Duration (time from first diagnosis until index date for prevalent cases, zero for incident cases), SMA subtype.
- Comorbidities evaluated during the 24 months prior to index: Charlson comorbidity index (CCI) score, and all comorbidities included within the CCI (myocardial infarction, congestive heart failure, peripheral vascular disease, cerebrovascular disease, dementia, chronic pulmonary disease, rheumatic disease, peptic ulcer disease, mild liver disease, diabetes without chronic complications, diabetes with chronic complications, hemiplegia or paraplegia, renal disease, malignancy, moderate or severe liver disease, metastatic solid tumour, and AIDS/HIV), anxiety and/or depression, and stress-related disorders.
- Active comedication evaluated during the three months prior to index: disease-modifying drugs, supportive care and other medications intended for the treatment of SMA symptoms or complications, drugs for anxiety and depression.
- Vital status evaluated at the end of follow-up: dead, alive; number of subjects in the reference cohort, censored due to death of SMA case to which they were matched, was also reported.

Outcomes, evaluated during the follow-up period:

- HCRU: outpatient visits, inpatient admissions, diagnostic and medical procedures, and surgical procedures were identified in the NPR. Diagnostic and medical procedures were identified using Klassifikation av vårdåtgärder (KVÅ) Medicinska (“Classification of care measures: medical”) codes and surgical procedures were identified using KVÅ Kirurgiska (“Classification of care measures: surgical”) codes [27]. Inpatient overnight stays involved at least one overnight stay, otherwise they were considered inpatient day cases.
- Length of stay (LOS): calculated as discharge date minus admission date for all hospitalisations with a least one overnight stay.
- Healthcare services received in the municipality identified in HSL: binary outcome for having received healthcare services per month of each year. These services include medical-related services that generally involve a home visit by a physician or nurse.
- Prescribed medication dispensed in community pharmacies: all dispensed medications (Anatomical Therapeutic Chemical [ATC] codes) recorded in the SPDR.
- Treatments used in the management of SMA symptoms identified using ATC codes in the SPDR: antibacterials for systemic use, opioids, other analgaesics and antipyretics, antidepressants, drugs for constipation, proton pump inhibitors, mucolytics, anxiolytics, antihistamines for systemic use, drugs for obstructive airway disease, and corticosteroids.
- Disease-modifying therapy (DMT): therapy and dates were identified in the NMiS. For patients not captured in the NMiS, a proxy using recordings of the procedure code DT012: "intrathecal administration of medicine" was built as data on medicines administered in-hospital is lacking in the SPDR and the NPR. Since nusinersen was the only DMT available during the study period and since nusinersen but not risdiplam or onasemnogene abeparvovec is administered intrathecally, it was assumed that all recordings of this procedure code were for nusinersen. This assumption was based on clinical expert advice and tested visually against data on actual nusinersen administration from the NMiS for the subset of patients covered in the registry.
- Direct medical costs: estimated for each patient by multiplying the number of occurrences of all healthcare services used as recorded in the NPR and HSL by the corresponding unit costs, as reported by the National Board of Health and Welfare (NBHW). The cost of nusinersen was calculated by multiplying the number of records by the public list price. For medications rendered in community pharmacies, the total cost (including amount reimbursed by the county council and patient co-payment, before VAT) that was recorded in SPDR was tallied for each patient. Costs were weighted with the tariffs published by the NBHW corresponding to the year of occurrence and then adjusted to the 2019 price level using the healthcare component of the Swedish Consumer Price Index. Costs were expressed in Swedish Krona (SEK) and converted to Euro (EUR) using the average conversion rate for 2021 [0.09856] [28, 29].

Individuals with missing age, gender or region were excluded as these were matching variables. Dates with missing day were imputed as the 15th of the month and dates with missing month were imputed as July. For the remaining variables the absence of a diagnostic or treatment code was taken to indicate absence of the disease/treatment.

### Statistical methods

Descriptive statistics (mean, standard deviation [SD] for continuous variables, number and proportion for categorical) were used to describe demographic and clinical characteristics of the SMA population by type compared with reference subjects at index. The number of subjects in the reference cohort, censored due to death of SMA case to which they were matched was also reported to contextualise this censoring event. Comparisons between SMA and corresponding reference cohorts were made using either Student’s *t*-test or the Mann–Whitney *U* test for continuous variables or the Chi-squared or Fisher’s exact test for categorical variables.

We modelled average annual HCRU and medical costs for SMA compared to the reference cohorts to estimate differences attributable to the disease (i.e., average treatment effect estimand). Rates and rate differences were generated to understand the intensity of use of each type of healthcare service per year. Annual risk estimates and unadjusted risk ratios (RR) were generated to understand the probability of individuals using healthcare services at least once per year. These values, along with corresponding 95% confidence intervals (CIs), for each type of healthcare service used by inpatients with SMA versus reference cohorts were estimated by using unadjusted generalised estimating equations (GEE) with the log-link function and negative binomial error distributions. Unadjusted mean number of bed days (i.e., LOS) and the difference in number of bed days were also estimated using GEE. During all follow-up, use of SMA medications and medications used to manage symptoms and/or complications were described in SMA cohorts only using number and proportion for all patients.

Mean direct medical costs were estimated by using a gamma regression model derived from GEE due to the right skew of the data. Although we wanted to adjust for all risk factors for HCRU at index only a limited number of these variables could be adjusted for in the analysis due to the small sample size and limited degrees of freedom. Therefore, the model was adjusted for age and sex and an exchangeable correlation matrix was used to account for correlations within individual patients over the years.

Since SMA is a rare disease, we needed to minimise sample depletion so all analyses included incident and prevalent subjects indexed at varying times in the course of their disease. The features of this study design impose left truncation and right censoring and prevent us from observing relevant data over each person’s full lifetime: prevalent SMA subjects are missing before 1 January 2007 and data for incident and prevalent SMA subjects are censored, at the latest, on 31 December 2019. Therefore, to account for left and right truncation we constructed synthetic cohorts [30]. Synthetic cohorts combine data from various individuals into cells corresponding to the time units that would be studied if a single cohort could be observed longitudinally. In the context of this study, data from patients with SMA and their matched non-SMA counterparts were aggregated, by computing means or proportions, into cells defined by years from the date of diagnosis (or the corresponding pseudo-diagnosis date for non-SMA individuals). Time since first SMA diagnosis was aggregated into yearly periods. Individuals only contributed to the numerator and denominator to the yearly units in which they had data. CIs were constructed using bootstrapping. The analyses were conducted in SAS version 9.4.

## Results

### Patient characteristics at index

We identified 290 patients with SMA and matched them to 1160 individuals from the general population (Table 1). Most patients with type 2 SMA, type 3 SMA, and UAO SMA were prevalent cases at index, while most patients with type 1 SMA were incident cases (Table 1).

**Table 1:** Demographic and clinical characteristics of patients with each SMA type and their respective reference cohort at index

	Type 1 SMA			Type 2 SMA			Type 3 SMA			UAO SMA		
	SMA *N* = 60	Reference *N* = 240	Standardised difference	SMA *N* = 55	Reference *N* = 220	Standardised difference	SMA *N* = 102	Reference *N* = 408	Standardised difference	SMA *N* = 73	Reference *N* = 292	Standardised difference
SMA status at index date, n (%)			0			0			0			0
Incident	52 (86.7)	208 (86.7)		25 (45.5)	100 (45.5)		49 (48.0)	196 (48.0)		25 (34.2)	100 (34.2)	
Prevalent	8 (13.3)	32 (13.3)		30 (54.5)	120 (54.5)		53 (52.0)	212 (52.0)		48 (65.8)	192 (65.8)	
Age at index, years			0			0			0			0
Mean (SD)	0.9 (2.0)	0.9 (2.0)		5.9 (5.3)	5.9 (5.3)		26.2 (22.2)	26.2 (22.1)		51.9 (16.1)	51.9 (16.0)	
Sex, n (%)			0			0			0			0
Male	26 (43.3)	104 (43.3)		34 (61.8)	136 (61.8)		52 (51.0)	208 (51.0)		48 (65.8)	192 (65.8)	
Female	34 (56.7)	136 (56.7)		21 (38.2)	84 (38.2)		50 (49.0)	200 (49.0)		25 (34.2)	100 (34.2)	
Duration since first symptom onset, years			0			0			0			0
Mean (SD)	0.5 (1.8)	0.5 (1.7)		3.7 (4.7)	3.7 (4.7)		3.5 (5.1)	3.5 (5.1)		2.8 (2.60)	2.8 (2.58)	
Median (p25–p75)	0 (0–0)	0 (0–0)		2.0 (0–6.0)	2.0 (0–6.0)		1.26 (0–5.2)	1.26 (0–5.2)		2.89 (0–4.8)	2.89 (0–4.8)	
Min to max	0–9.8	0–9.8		0–19.7	0–19.7		8.34–20.0	8.34–20.0		0–9.8	0–9.8	
Active comedications in SMA population (identified 3 months before, and excluding index date)												
Nusinersen	0 (0)	0 (0)	0	0 (0)	0 (0)	0	0 (0)	0 (0)	0	0 (0)	0 (0)	0
Supportive care drugs^a^	7 (11.7)	8 (3.3)	0.320	13 (23.6)	7 (3.2)	0.6293	7 (6.9)	15 (3.7)	0.1430	7 (9.6)	23 (7.9)	0.0607
Other medications intended for the treatment of SMA^b^	0 (0)	0 (0)	NE^*^	0 (0)	0 (0)	NE^*^	n < 5	0 (0)	0.1407	0 (0)	0 (0)	NE^*^
Drugs against anxiety or depression	0 (0)	0 (0)	NE^*^	0 (0)	0 (0)	NE^*^	n < 5	6 (1.5)	0.0446	5 (6.8)	10 (3.4)	0.1556
Pre-existing mental health conditions (identified during all available time prior to index)												
Anxiety and/or depression	0 (0)	0 (0)	NE^*^	0 (0)	n < 5	− 0.0956	11 (10.8)	27 (6.6)	0.1482	22 (30.1)	47 (16.1)	0.3378
Stress-related disorders	0 (0)	0 (0)	NE^*^	0 (0)	0 (0)	NE^*^	0 (0)	n < 5	− 0.0993	n < 5	8 (2.7)	0.0754
Vital status at the end of study follow-up, n (%)												
Death	43 (71.7)	0 (0)	2.2492	6 (10.9)	0 (0)	0.4949	9 (8.8)	15 (3.7)	0.2138	15 (20.5)	24 (8.2)	0.3569
Alive	17 (28.3)	68 (28.3)	0	49 (89.1)	196 (89.1)	0	93 (91.2)	363 (89.0)	0.0738	58 (79.5)	219 (75.0)	0.1063

Presented comorbidities only for conditions where count was above 5. The full results with the rest of the comorbidities can be found in Supplementary Table 1. a Supportive care medications include: drugs for functional gastrointestinal disorders, drugs for constipation, antibacterials for systemic use, antihistamines for systemic use, corticosteroids for systemic use, opioids, other analgesics and antipyretics, anabolic steroids, drugs for obstructive airway disease, proton pump inhibitors, mucolytics; b Other medications include: riluzole, valproic acid, hydroxycarbamide, sodium phenylbutyrate, glycerol phenylbutyrate, phosphocreatine, acetylcarnitine, levocarnitine, somatropin probenecid

*NE* not estimable; *SD* standard deviation; *SMA* spinal muscular atrophy; *UAO* unclassified adult onset

The mean (SD) duration since first symptom onset ranged from 0.5 (1.8) years for patients with type 1 SMA to 3.7 (4.7) years for type 2 SMA (Table 1). Mean (SD) age at index ranged from 0.8 (1.8) years in patients with type 1 SMA to 51.6 (16.1) years in UAO SMA (Table 1). Most patients with type 1 SMA were female, while most patients with type 2 SMA, type 3, and UAO SMA were male (Table 1).

The prevalence of comorbidities in the Charlson comorbidity score did not differ (based on standardised mean difference) between any of the SMA subtypes and their respective reference cohorts (Supplementary Table 1) except for myocardial infarction, cerebral vascular disease, hemiplegia or paraplegia, and renal disease which were all higher in SMA patients compared with reference cohorts. Significant differences were observed in patients with type 1 SMA and type 2 SMA who used more supportive care drugs at index than their reference cohort. In addition, patients with UAO SMA were more likely to have had a history of anxiety or depression at index than their reference cohort (Table 1).

By the end of follow-up, proportionately more patients with type 1, type 3, and UAO SMA had died than their reference cohorts (Table 1).

### HCRU during follow-up

#### Use of HCRU (assessed using rate and rate difference)

When comparing across SMA types using the unadjusted annual rate, those with type 1 generally had the highest consumption of all resources, except for consumption of prescribed medications dispensed in the outpatient setting and healthcare received in the municipality, which tended to be highest for patients with UAO SMA (Supplementary Table 2).

**Fig. 1:**
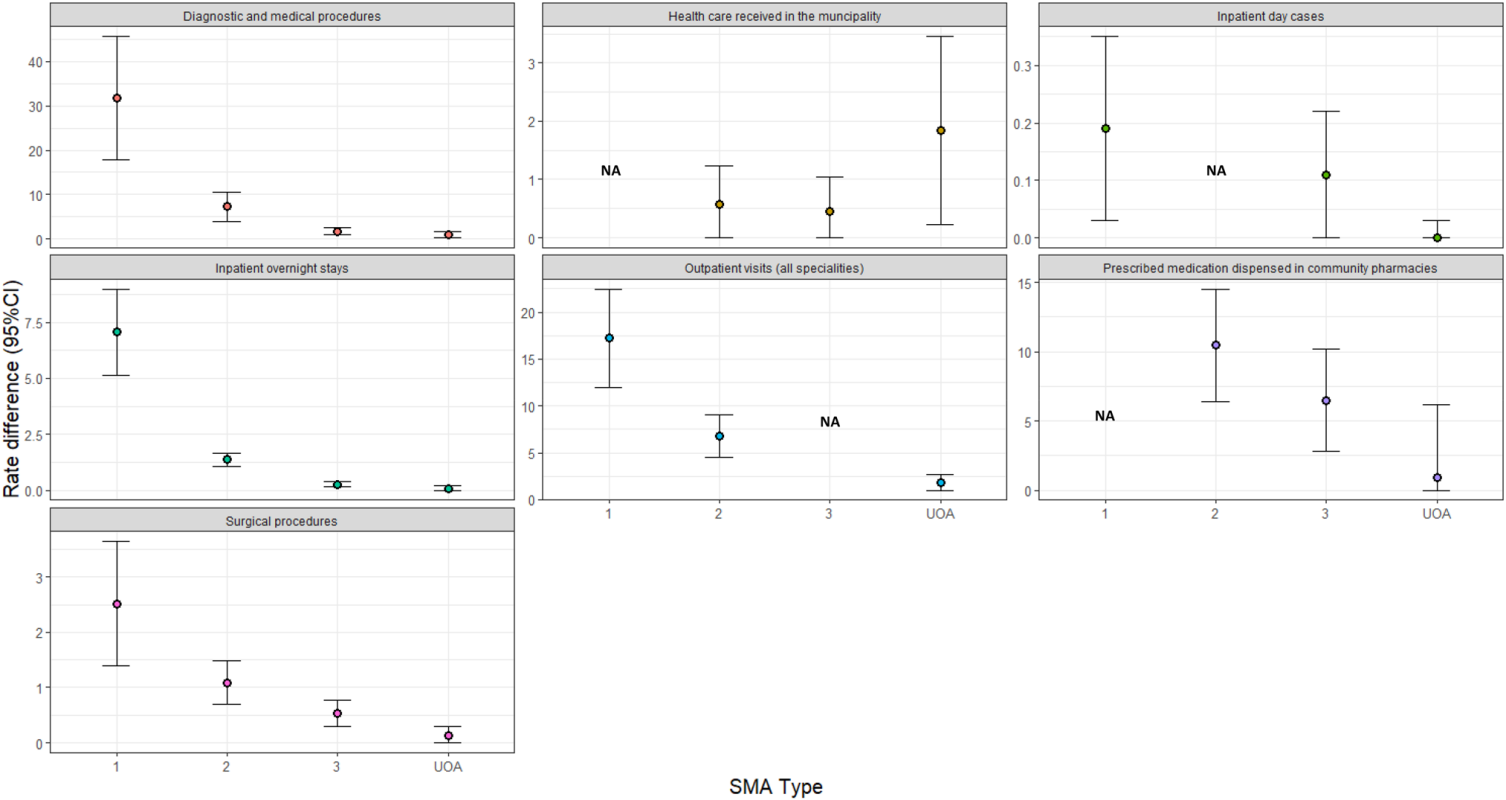
Rate difference of HCRU in SMA subtypes versus respective reference cohorts during follow-up. Abbreviations: CI = confidence interval; NA = not applicable; SMA = spinal muscular atrophy; UAO = unspecified adult onset. Note: for all of the models we assumed the same distribution and covariance matrix structure and in some cases this was not a good fit and the model would not converge, which is why there are NA for some outcomes

When comparing SMA patients with the reference cohorts using the rate difference, patients with SMA generally had a higher consumption of all HCRU during the follow-up period than their counterparts. The greatest difference in HCRU (i.e., rate difference) compared with reference cohorts was for diagnostic and medical procedures for patients with type 1 SMA and the second highest was for outpatient visits for patients with type 1 SMA (Fig. 1 and Supplementary Table 2).

#### Likelihood of HCRU (assessed using risk and risk ratio)

When comparing across SMA types using the unadjusted risk, patients with type 2 SMA tended to have the highest likelihood of having an outpatient visit, followed by a prescription dispensed in the outpatient setting. The third highest was for patients with type 1 who tended to have higher likelihood of an outpatient visit (Supplementary Table 2).

**Fig. 2:**
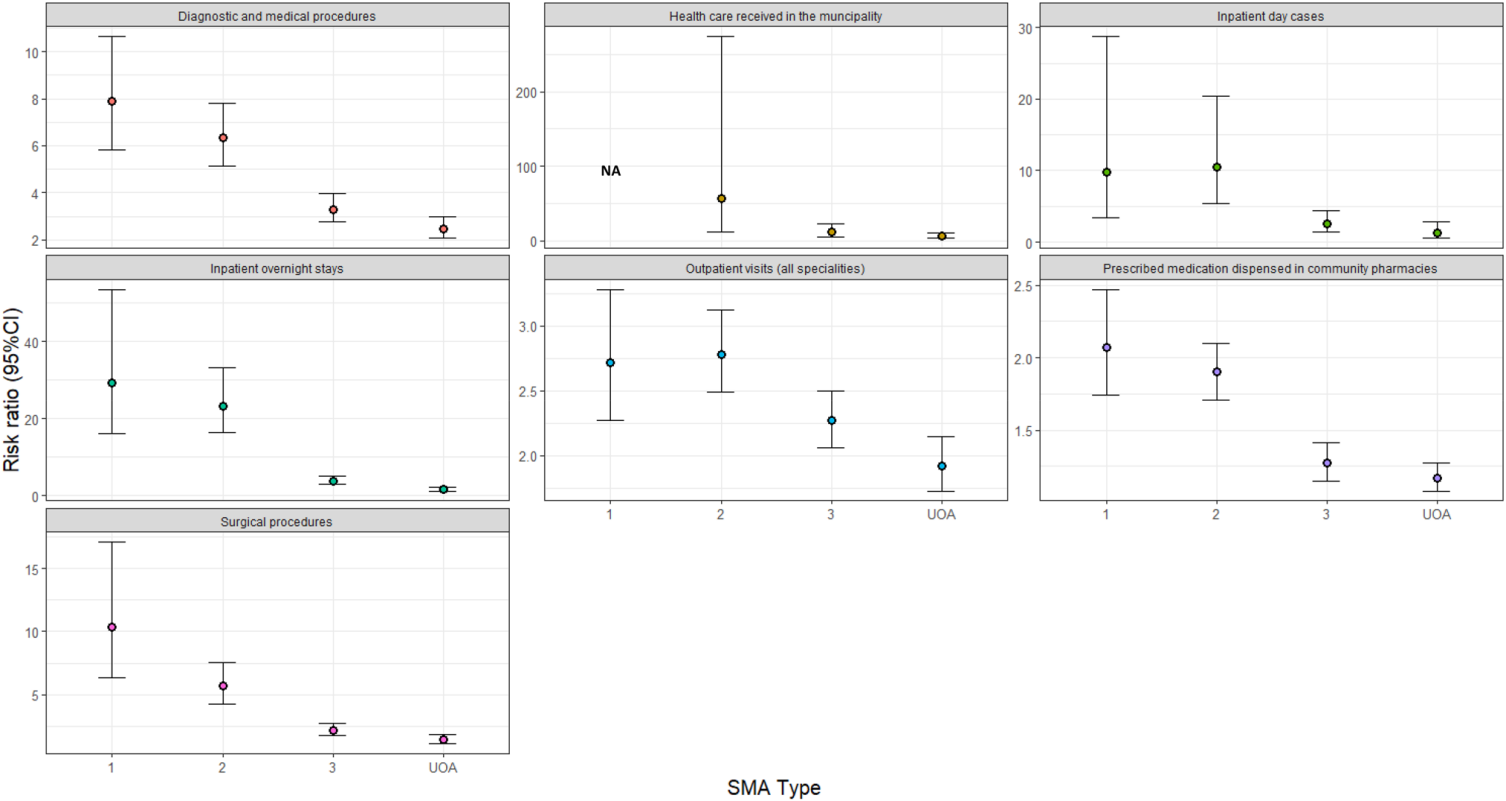
Unadjusted risk ratio of HCRU in SMA subtypes versus respective reference cohorts during follow-up. Abbreviations: CI = confidence interval; NA = not applicable; SMA = spinal muscular atrophy; UAO = unspecified adult onset.. Note: for all of the models we assumed the same distribution and covariance matrix structure and in some cases this was not a good fit and the model would not converge, which is why there are NA for some outcomes

When comparing SMA with reference cohorts using the risk ratio, patients with SMA were generally more likely to have used more healthcare services during the study follow-up period compared with reference cohorts (Fig. 2, Supplementary Table 2). The greatest difference in the likelihood of use (i.e., unadjusted RR) was for healthcare received in the municipality for patients with type 2 SMA (although with a high degree of variation in this difference). The next greatest differences between SMA and reference cohorts were for inpatient overnight stays; patients with type 1 SMA and type 2 SMA showed more than 20-fold higher risk of admission than their reference counterparts (Figs. 1, 2 and Supplementary Table 2). Amongst those with at least one overnight stay, the LOS was longer for patients with type 1 and type 2 SMA, compared with the corresponding references (Supplementary Table 2).

#### SMA management during follow-up

**Fig. 3:**
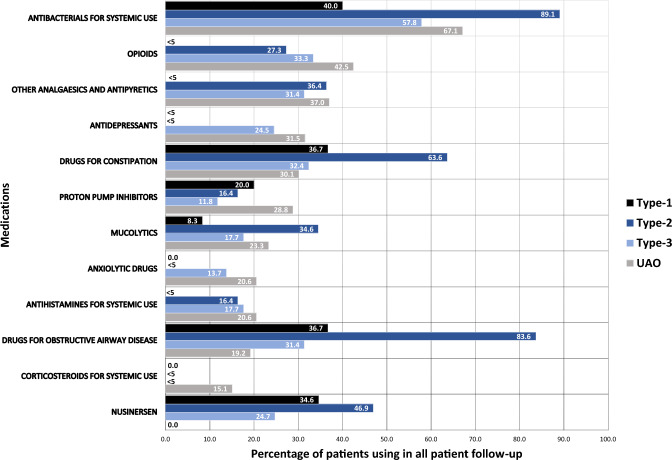
Use of SMA-related medications in all patients during follow-up. Abbreviation: UAO = unspecified adult onset. Note: cells < 5 cannot be displayed in line with Statistics Sweden policy. The denominator for nusinersen is 26 for type 1, 49 for type 2, 97 for type 3, and 0 for UAO because the first intrathecal code had to occur < 18 years of age i.e., when they were eligible for nusinersen in Sweden at the time of data analysis. Follow-up was different for different subtypes (see Table 1), which should be taken into consideration when comparing across the cohorts

During patient follow-up, nearly one-third of the patients with type 1 SMA used systemic antibacterials, drugs for constipation and obstructive airway problems, and nusinersen (Fig. 3 and Supplementary Table 3). More than four in five patients with SMA type 2 used systemic antibacterials and drugs for obstructive airway problems; about two-thirds used drugs for constipation and about half used nusinersen. More than half of the patients with type 3 SMA used systemic antibacterials, and one-third of them used opioids, drugs for constipation, other analgaesics and antipyretics, or drugs for obstructive airway problems; about one in four patients with type 3 SMA used nusinersen or antidepressants. Among patients with UAO SMA, nearly two-thirds used systemic antibacterials, nearly half of them used opioids, about one-third used other analgaesics and antipyretics, antidepressants or drugs for constipation, while about one in five used anxiolytic drugs (Fig. 3 and Supplementary Table 3).

### Trajectory of HCRU since SMA diagnosis

For patients with type 2 SMA, the number of overnight inpatient stays and medications dispensed in the outpatient setting were higher than in the reference cohort in each year since SMA diagnosis. For patients with all other SMA subtypes, HCRU was different in some years, but similar in other years when compared to the respective reference cohorts (Supplementary Figs. 1–5).

### Trajectory of direct medical costs

For all SMA cohorts, annual average direct medical cost attributable to SMA since first SMA diagnosis was higher than in the respective reference cohorts. This cost (which includes costs of nusinersen from 2017) was greatest for patients with type 1 SMA, followed by patients with type 2 SMA, type 3 SMA, and UAO (Table 2).

**Table 2:** Total average annual direct medical costs during follow-up by SMA type compared with reference cohorts*

	SMA cohort	Reference cohort
*Type 1 SMA*		
Total mean predicted per patient annual medical cost of care (95% CI)	SEK 1,158,528 (663,955–1,653,101) €114,185 (€65,439–€162,930)	SEK 8160 (3863–12,457) €804 (€381–€1228)
The difference in mean predicted per patient direct medical cost (95% CI)	SEK 1,150,368 (657,753–1,642,982) €113,380 (€64,828, €161,932)	
*Type 2 SMA*		
Total mean predicted per patient annual medical cost of care (95% CI)	SEK 627,805 (396,000–859,609) €61,876 (€39,030–€84,723)	SEK 6492 (3891–9094) €640 (€383–€896)
Difference in mean predicted per patient direct medical cost (95% CI)	SEK 621,312 (390,240–852,384) €61,237 (€38,462–€84,011)	
*Type 3 SMA*		
Total mean predicted per patient annual medical cost of care (95% CI)	SEK 461,826 (251,137–672,514) €45,518 (€24,752–€66,283)	SEK 9757 (7219–12,295) €962 (€711–€1212)
Difference in mean predicted per patient direct medical cost (95% CI)	SEK 452,069 (241,325–662,813) €44,556 (€23,785–€65,327)	
*UAO SMA*		
Total mean predicted per patient annual medical cost of care (95% CI)	SEK 41,051 (30,660–51,441) €4046 (€3022–€5070)	SEK 19,766 (15,301–24,230) €1948 (€1508–€2388)
The difference in mean predicted per patient direct medical cost (95% CI)	SEK 21,285 (10,465–32,105) €2098 (€1031–€3164)	

*CI* confidence interval; *SEK* Swedish krona; *SMA* spinal muscular atrophy; *UAO* unclassified adult onset

SEK was converted to EUR using the average conversion rate for 2021

*Costs for nusinersen were included in the cost calculation, however, since this therapy was only approved in 2017, the cost of these medications will not be fully realised

**Fig. 4:**
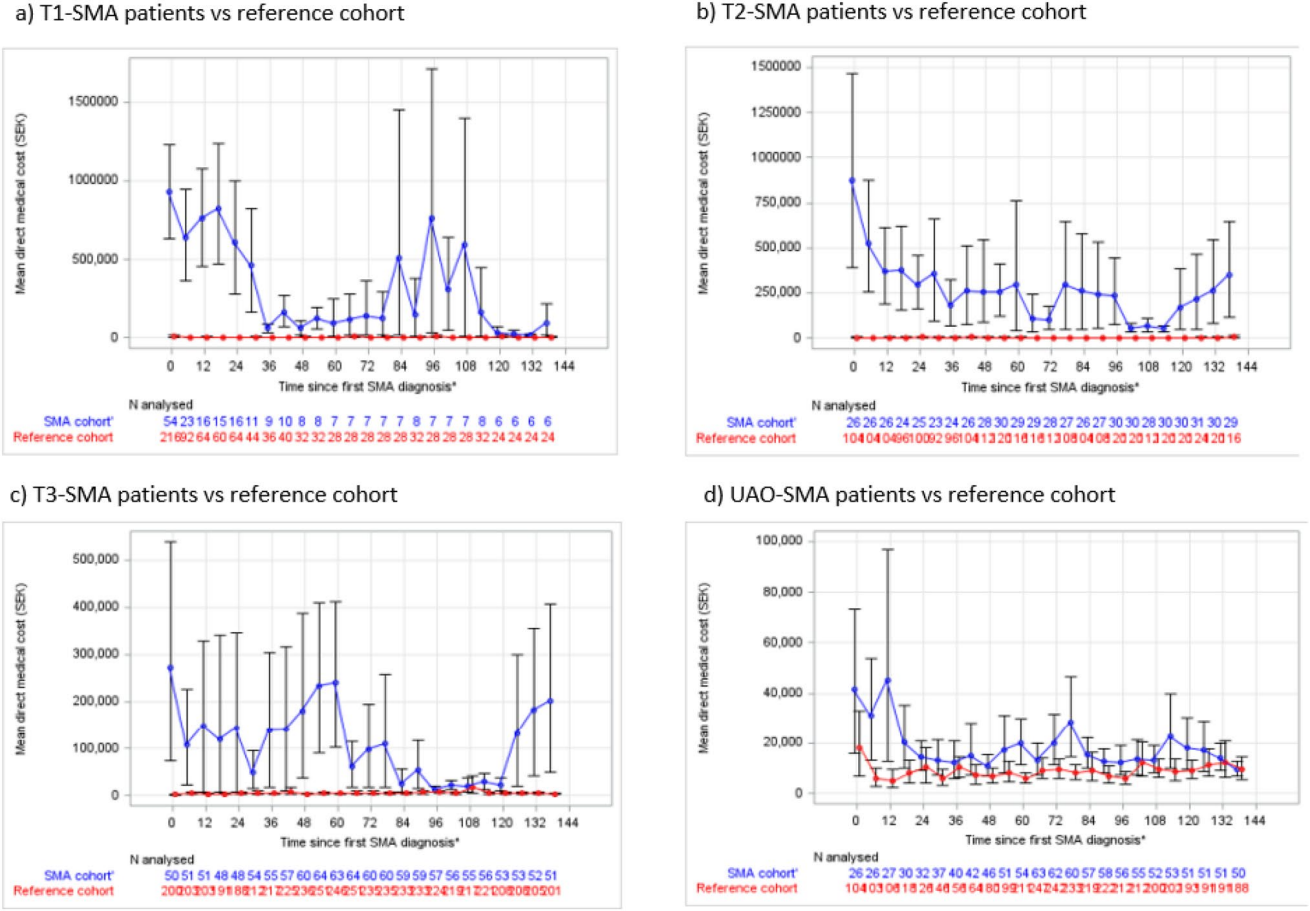
The evolution of direct medical costs over time since first diagnosis. **a** Type 1 SMA patients vs reference cohort; **b** type 2 SMA patients vs reference cohort; **c** type 3 SMA patients vs reference cohort; **d** unspecified adult onset (UAO) SMA patients vs reference cohort. Abbreviations: CI = confidence interval; SEK = Swedish krona; SMA = spinal muscular atrophy. 95% CIs constructed using bootstrapping methods. Note: since these are synthetic cohorts, individuals can enter and leave the annual cohorts each year, therefore, the numbers at risk fluctuate between the years

Direct medical costs were greatest in the 2–3 years after the first diagnosis for all SMA subtypes; however, direct medical costs were significantly higher for patients with type 2 SMA than for the reference cohorts for all times since their first SMA diagnosis. For all other SMA subtypes, the point estimates showed that there were greater direct medical costs for all times since their first SMA diagnosis; however, there was some overlap in CIs in some years (Fig. 4).

## Discussion

The current study assessed HCRU and direct medical costs among patients with SMA, by subtype, and compared them to those of matched-reference cohorts from the general population in Sweden. Overall, clinical characteristics were similar at index between SMA patients and their references. During follow-up, the HCRU and direct medical costs incurred in the care of patients with SMA (regardless of the subtype) were significantly higher than those without SMA (reference cohorts). HCRU rates were highest for patients with type 1 SMA across most of the healthcare resources categories (outpatient specialist visits, inpatient day, overnights stays and associated length of stays, surgeries, and diagnostic and medical procedures), followed by patients with type 2 SMA. However, healthcare received in the municipality and prescriptions dispensed in the outpatient setting, were highest for UAO SMA patients.

Our finding indicating higher HCRU by patients with type 1 SMA is expected given that these patients require significant upfront care due to the severity of their condition. This is also consistent with previous literature reviews reporting higher resource use by these patients [16–18, 20–22]. One survey conducted in Germany by Klug et al. [14], demonstrated that across SMA types 1 to 3, the highest resource use was amongst types 1 and 2.

Over the course of the entire follow-up, use of antidepressant, anxiolytics, or opioid medications were prominent in those with type 3 SMA or UAO SMA. These findings are equally not surprising given that these patients typically experience chronic pain and stress [31]. These findings are also consistent with the Klug et al. study that reported use of psychotropic medications to be most prominent in patients with type 3 SMA [14]. Most previous studies did not report HCRU by SMA subtypes or only reported for patients with type 1 to 3 [16–18]. Therefore, to our knowledge, the current study is unique as it not only provides updated estimates of HCRU, but it also provides those estimates by each SMA type, including resources used outside the hospital for patients with UAO SMA. Those with adult symptom onset are generally considered to have the mildest type [32]; however, clearly these individuals are still burdened, although the burden appears to be of a different nature. SMA patients with subtype 3 or with adult onset should therefore be considered in health economic assessments of SMA treatments. In addition, this is the first known study to date [17] that has reported on the use of diagnostic, medical, and surgical procedures in patients with SMA. As evidenced by our findings, these healthcare resources were among the most commonly used services in SMA types 1 and 2, and as such, they should be included in future assessments of HCRU among patients with SMA [1, 7, 14, 17]. The study is also unique as it presents HCRU for each year since first SMA diagnosis by subtype, which allows the progressive nature of the disease to be realised.

Annual direct medical costs were higher for all SMA subtypes compared with reference cohorts from the general population. In particular, the highest direct medical costs involved type 1 SMA (€114,185), followed by type 2 SMA (€61,876), type 3 SMA (€45,518), and UAO SMA (€4046). While studies from around the world report varying cost estimates due to differences in cost assessment methods, geographies, data sources, and time periods, our findings on average annual costs by SMA type are generally aligned to those previously reported [18]. For example, the direct medical cost of €114,185 in patients with type 1 SMA is within the range of previous estimates (€59,480.77 [Germany, 2013 estimate at 2021 prices] [14, 19] to €172,346 [Australia, 2017 estimate at 2021 prices] [19, 33]). More recently, a study conducted in Canada reported a mean direct annual cost per patient of €33,277 [at 2021 prices] for those with a first diagnosis < 6 months of age, €36,412 [at 2021 prices] for those first diagnosed at 6 months to < 18 months of age, and €13,433 [at 2021 prices] in those aged ≥ 18 months to < 18 years at first diagnosis [21]. However, these recent cost estimates from Canada cannot be generalised to European countries given the differences in healthcare systems. Hence, the current study provides crucial information on updated direct medical costs per each SMA type in Europe.

### Study limitations

A key limitation of the study is that visits to primary care were not assessed. There is no national primary care registry in Sweden. Primary care data in Sweden is currently only available for research for three regions, i.e., separate regional databases in Stockholm, Västra Götaland, and Skåne, corresponding to around 52% of the Swedish population [34]. To maximize patient counts and minimize project complexity, primary care data were not included as potential linkable datasets in the current study. Primary care could be partially inferred through prescriptions dispensed in the outpatient setting, but the full burden of this resource could not be assessed. Additionally, the price for nusinersen (the only DMT available during the study period) was based on public list price since the discounted price is not publicly available. The cost of DMTs in the current study is therefore likely to be underestimated. However, since nusinersen was introduced in Sweden late in the study period (2017 onwards) and since costs are averaged over the study period, the increased cost due to nusinersen will not be fully realised. The study needs to be repeated in a time period when sufficient follow-up data after DMT approval has been accrued.

Although the SMA sample size for this study may appear small the nature of the data ensured that longitudinal data for up to 22 years was collected so that the progressive nature of the disease could be fully realised. Furthermore, another strength of this study is the use of disease registry (NMiS) linked to other patient registries for assessing SMA subtype. A recent literature review focusing on studies on cost and HCRU associated with SMA summarised that use of disease registry networks could play an important role in addressing the current gaps in literature related to resource use and costs [17].

## Conclusions

This study demonstrated that the HCRU and direct medical costs represent a significant burden on patients with SMA of all subtypes as well as on the Swedish healthcare system. The highest HCRU and direct medical costs were incurred in the care of patients with type 1 SMA, followed by type 2 SMA and other subtypes. For patients with adult symptom onset SMA, the burden of disease may be of a different nature, which is exemplified by the elevated levels of antidepressant, anxiolytics, and opioid medications. Further research is needed to understand the healthcare and economic burden in other European countries, as well as with further advancements in new treatments over time.

## Supplementary Information

Below is the link to the electronic supplementary material.

Supplementary file1 (DOCX 1078 KB)

## Data Availability

The data that support the findings of this study are available from Socialstyrelsen and The Swedish National Registry for Neuromuscular Disorders. Restrictions apply to the availability of these data, which were used under license for this study. Data are available from https://www.socialstyrelsen.se/en/ and https://www.neuroreg.se/en/neuromuskulara-sjukdomar-nmis/.
