# Supplementary material for: Healthcare resource utilisation and direct medical cost for individuals with 5q spinal muscular atrophy in Sweden
Source: Eur J Health Econ. 2024 Apr 20;26(1):35–48. doi: 10.1007/s10198-024-01678-y (PMC11743398; doi:10.1007/s10198-024-01678-y)
Supplement: Supplementary file 1 — Supplementary file1 (DOCX 1078 KB) [file 10198_2024_1678_MOESM1_ESM.docx]

# Supplementary Figures

**Supplementary Figure 1. The evolution of outpatient visits over time since first diagnosis**. a) Type 1 SMA patients vs reference cohort; b) Type 2 SMA patients vs reference cohort; c) Type 3 SMA patients vs reference cohort; d) Unspecified adult onset (UAO) SMA patients vs reference cohort.


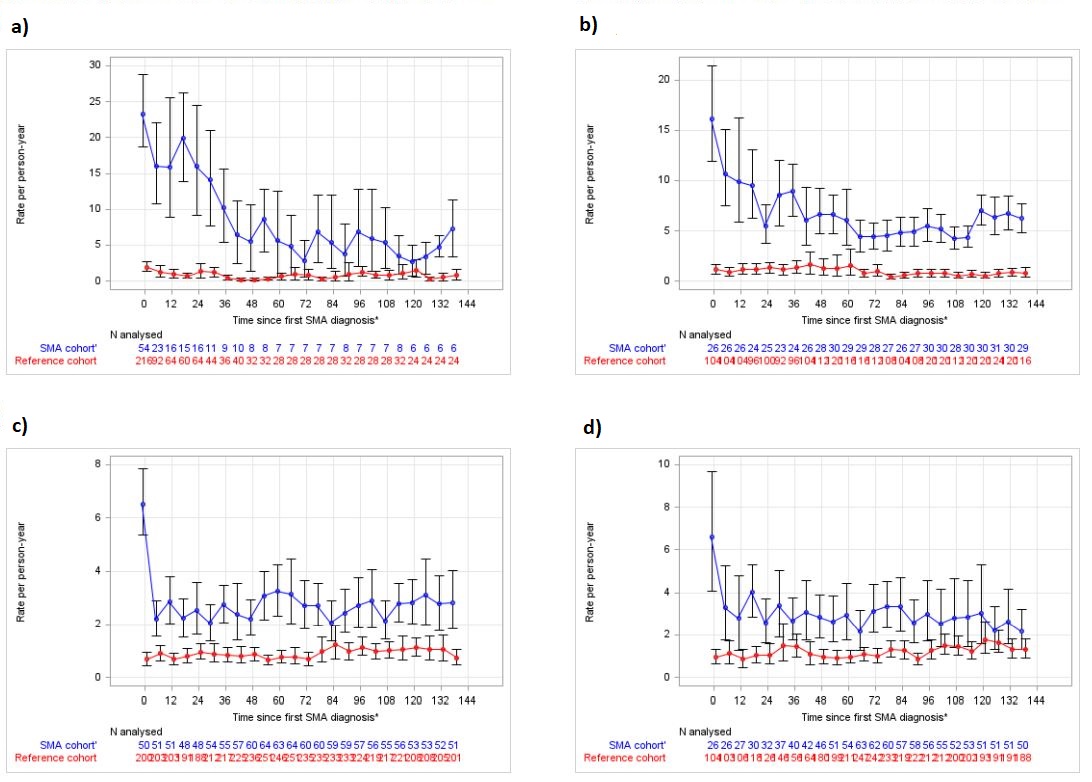


Abbreviation: SMA = spinal muscular atrophy. 95% CIs constructed using bootstrapping methods.

Note: since these are synthetic cohorts, individuals can enter and leave the annual cohorts each year. Therefore, the numbers at risk fluctuate between the years.

*Time from first SMA diagnosis until resource use.

**Supplementary Figure 2. The evolution of inpatient overnight stays over time since first diagnosis.** a) Type 1 SMA patients vs reference cohort; b) Type 2 SMA patients vs reference cohort; c) Type 3 SMA patients vs reference cohort; d) Unspecified adult onset (UAO) SMA patients vs reference cohort.


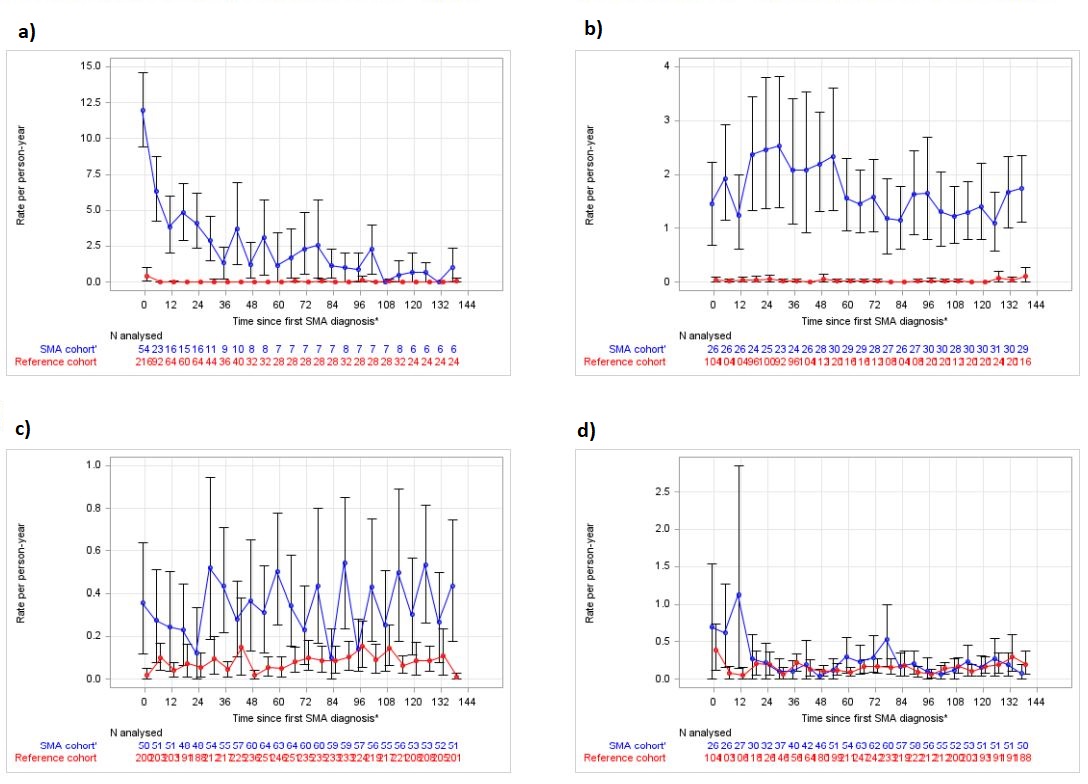


Abbreviation: SMA = spinal muscular atrophy. 95% CIs constructed using bootstrapping methods.

Note: since these are synthetic cohorts, individuals can enter and leave the annual cohorts each year, therefore, the numbers at risk fluctuate between the years.

*Time from first SMA diagnosis until resource use.

**Supplementary Figure 3. The evolution of diagnostic and medical procedures over time since first diagnosis.** a) Type 1 SMA patients vs reference cohort; b) Type 2 SMA patients vs reference cohort; c) Type 3 SMA patients vs reference cohort; d) Unspecified adult onset (UAO) SMA patients vs reference cohort.


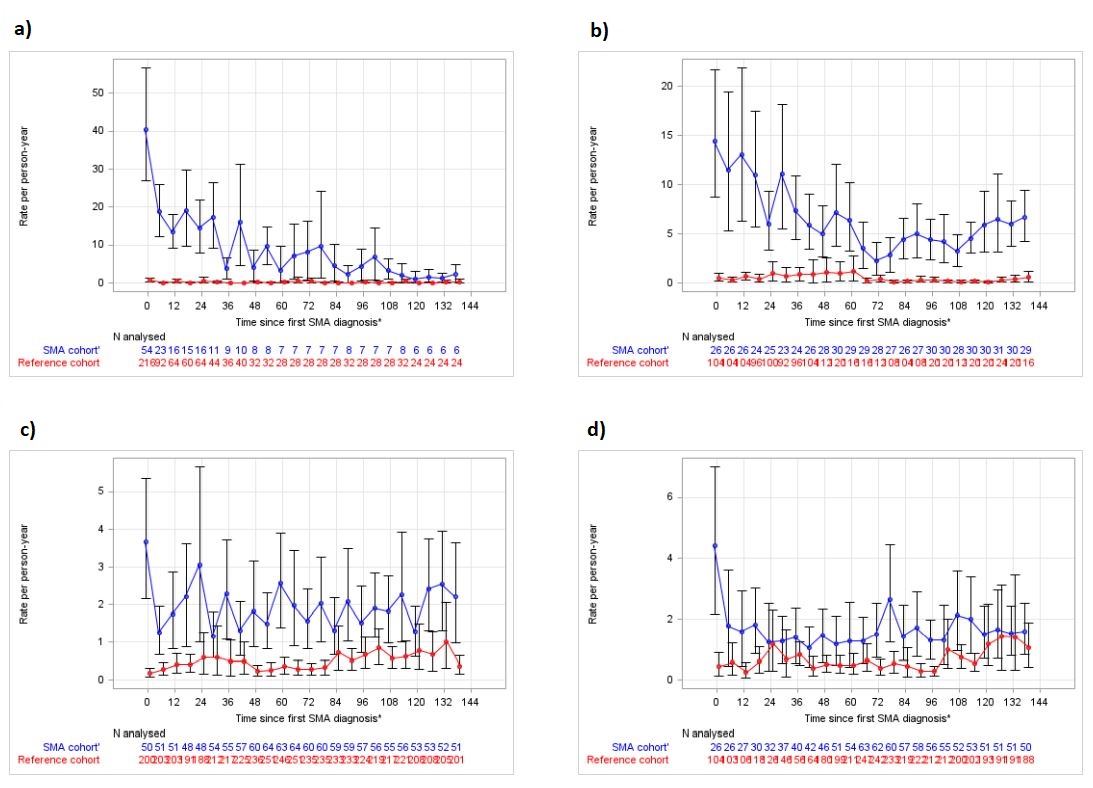


Abbreviation: SMA = spinal muscular atrophy. 95% CIs constructed using bootstrapping methods.

Note: since these are synthetic cohorts, individuals can enter and leave the annual cohorts each year, therefore, the numbers at risk fluctuate between the years.

*Time from first SMA diagnosis until resource use.

**Supplementary Figure 4. The evolution of surgical procedures over time since first diagnosis.** a) Type 1 SMA patients vs reference cohort; b) Type 2 SMA patients vs reference cohort; c) Type 3 SMA patients vs reference cohort; d) Unspecified adult onset (UAO) SMA patients vs reference cohort.


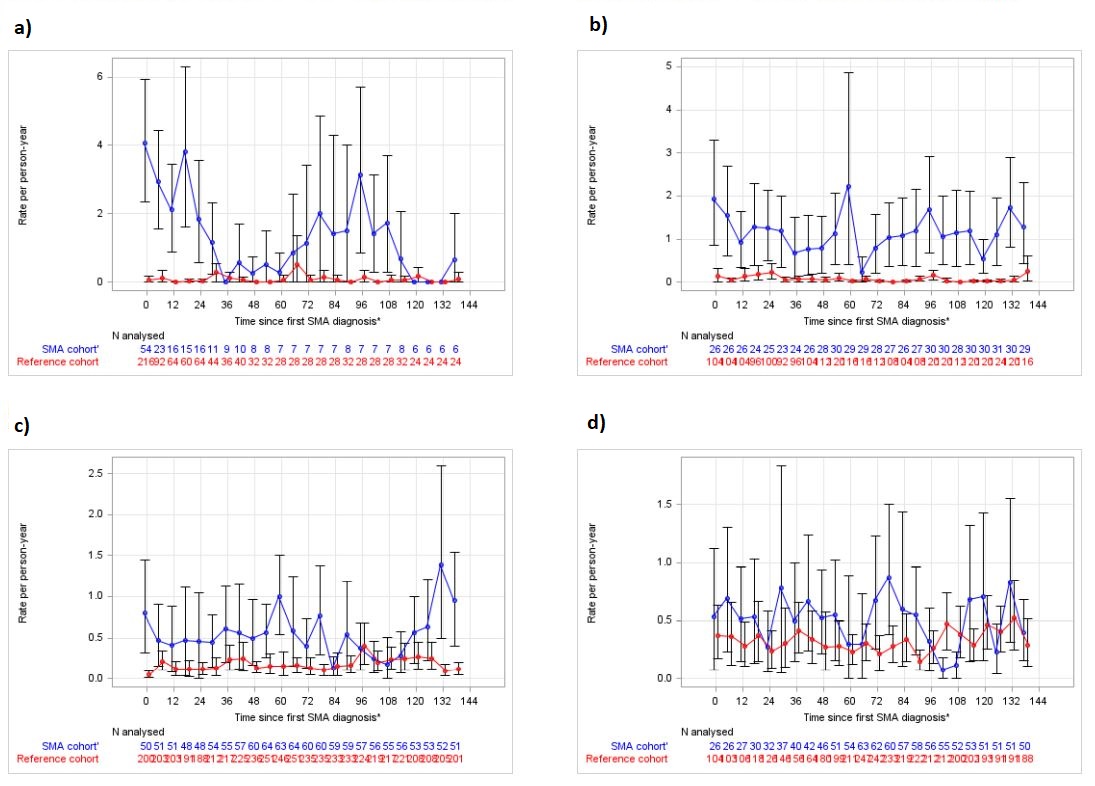


Abbreviation: SMA = spinal muscular atrophy. 95% CIs constructed using bootstrapping methods.

Note: since these are synthetic cohorts, individuals can enter and leave the annual cohorts each year, therefore, the numbers at risk fluctuate between the years.

*Time from first SMA diagnosis until resource use.

**Supplementary Figure 5. The evolution of prescriptions dispensed in the outpatient setting over time since first diagnosis.** a) Type 1 SMA patients vs reference cohort; b) Type 2 SMA patients vs reference cohort; c) Type 3 SMA patients vs reference cohort; d) Unspecified adult onset (UAO) SMA patients vs reference cohort.


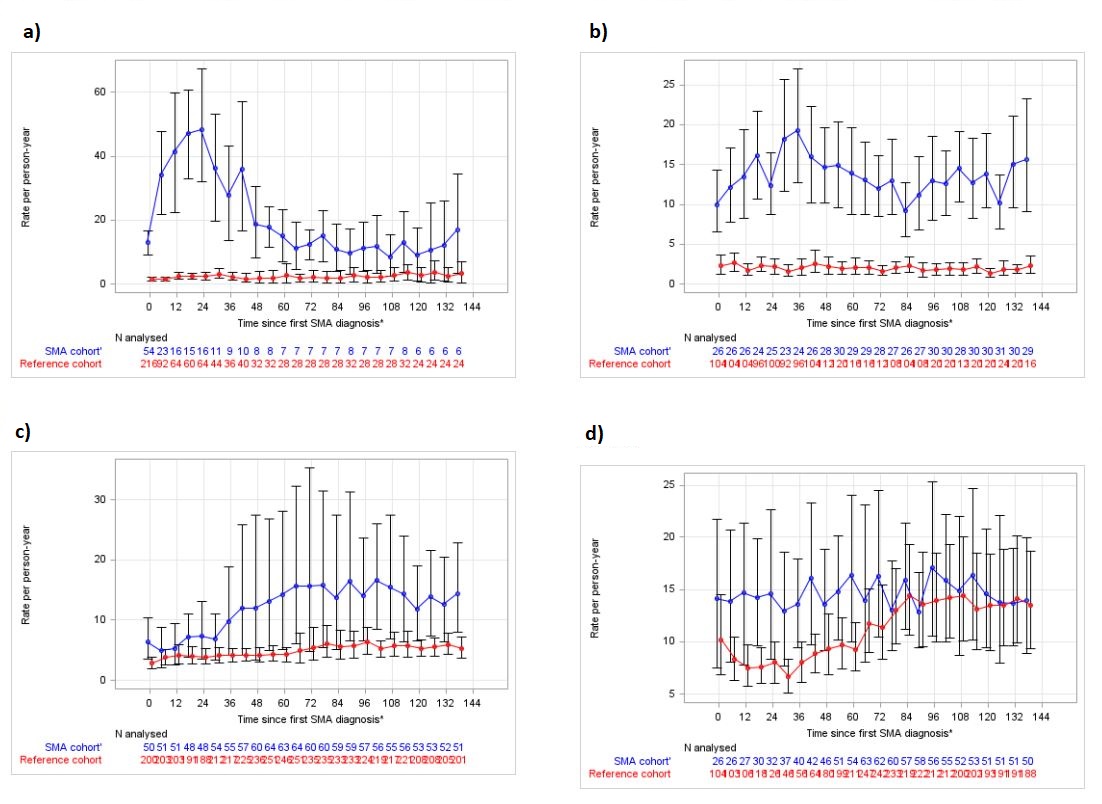


Abbreviation: SMA = spinal muscular atrophy. 95% CIs constructed using bootstrapping methods.

Note: since these are synthetic cohorts, individuals can enter and leave the annual cohorts each year, therefore, the numbers at risk fluctuate between the years.

*Time from first SMA diagnosis until resource use.

# Supplementary Tables

**Supplementary Table 1. Comorbidities in the CCI score at index among patients with SMA and reference cohorts**

| **Charlson comorbidities (during pre-index period)** | **Type 1 SMA** | | | **Type 2 SMA** | | | **Type 3 SMA** | | | **UAO SMA** | | |
| --- | --- | --- | --- | --- | --- | --- | --- | --- | --- | --- | --- | --- |
|  | **SMA** | **References** | **Standardised difference** | **SMA** | **References** | **Standardised difference** | **SMA** | **References** | **Standardised difference** | **SMA** | **References** | **Standardised difference** |
| Myocardial infarction | 0 (0.0%) | 0 (0.0%) | NE | 0 (0.0%) | 0 (0.0%) | NE | 0 (0.0%) | n < 5 | -0.0701 | n < 5 | 0 (0.0%) | 0.2374 |
| Congestive heart failure | 0 (0.0%) | 0 (0.0%) | NE | 0 (0.0%) | 0 (0.0%) | NE | 0 (0.0%) | 0 (0.0%) | NE | n < 5 | n < 5 | 0.0680 |
| Peripheral vascular disease | 0 (0.0%) | 0 (0.0%) | NE | 0 (0.0%) | 0 (0.0%) | NE | 0 (0.0%) | 0 (0.0%) | NE | 0 (0.0%) | n < 5 | -0.0829 |
| Cerebrovascular disease | 0 (0.0%) | 0 (0.0%) | NE | 0 (0.0%) | 0 (0.0%) | NE | n < 5 | n < 5 | 0.0943 | n < 5 | 0 (0.0%) | 0.1667 |
| Dementia | 0 (0.0%) | 0 (0.0%) | NE | 0 (0.0%) | 0 (0.0%) | NE | 0 (0.0%) | n < 5 | -0.0701 | 0 (0.0%) | n < 5 | -0.0829 |
| Chronic pulmonary disease | 0 (0.0%) | n < 5 | -0.1841 | n < 5 | 8 (3.6%) | 0 | 0 (0.0%) | 7 (1.7%) | -0.1868 | 0 (0.0%) | 6 (2.1%) | -0.2048 |
| Rheumatic disease | 0 (0.0%) | 0 (0.0%) | NE | 0 (0.0%) | 0 (0.0%) | NE | 0 (0.0%) | n < 5 | -0.0993 | n < 5 | n < 5 | 0.1262 |
| Peptic ulcer disease | 0 (0.0%) | 0 (0.0%) | NE | 0 (0.0%) | 0 (0.0%) | NE | 0 (0.0%) | 0 (0.0%) | NE | 0 (0%) | n < 5 | -0.1174 |
| Mild liver disease | 0 (0.0%) | 0 (0.0%) | NE | 0 (0.0%) | 0 (0.0%) | NE | 0 (0.0%) | 0 (0.0%) | NE | 0 (0%) | n < 5 | -0.1667 |
| Diabetes without chronic complications | 0 (0.0%) | 0 (0.0%) | NE | 0 (0.0%) | 0 (0.0%) | NE | n < 5 | n < 5 | 0.0574 | n < 5 | n < 5 | 0.1262 |
| Diabetes with chronic complications | 0 (0.0%) | 0 (0.0%) | NE | 0 (0.0%) | 0 (0.0%) | NE | 0 (0.0%) | n < 5 | -0.0701 | n < 5 | n < 5 | 0 |
| Hemiplegia or paraplegia | 0 (0.0%) | 0 (0.0%) | NE | n < 5 | 0 (0.0%) | 0.1925 | 0 (0.0%) | n < 5 | -0.0993 | n < 5 | n < 5 | 0.1117 |
| Renal disease | 0 (0.0%) | 0 (0.0%) | NE | 0 (0.0%) | 0 (0.0%) | NE | 0 (0.0%) | n < 5 | -0.0701 | n < 5 | 0 (0.0%) | 0.2374 |
| Malignancy | 0 (0.0%) | 0 (0.0%) | NE | 0 (0.0%) | 0 (0.0%) | NE | n < 5 | n < 5 | 0.1064 | 6 (8.2%) | 10 (3.4%) | 0.2058 |
| Moderate or severe liver disease | 0 (0.0%) | 0 (0.0%) | 0 | 0 (0.0%) | 0 (0.0%) | 0 | 0 (0.0%) | 0 (0.0%) | 0 | 0 (0.0%) | 0 (0.0%) | 0 |
| Metastatic solid tumour | 0 (0.0%) | 0 (0.0%) | NE | 0 (0.0%) | 0 (0.0%) | NE | 0 (0.0%) | 0 (0.0%) | NE | 0 (0.0%) | n < 5 | -0.1174 |
| AIDS/HIV | 0 (0.0%) | 0 (0.0%) | 0 | 0 (0.0%) | 0 (0.0%) | 0 | 0 (0.0%) | 0 (0.0%) | 0 | 0 (0.0%) | 0 (0.0%) | 0 |

Abbreviations: AIDS = acquired immunodeficiency syndrome; HIV = human immunodeficiency virus; SMA = spinal muscular atrophy.

**Supplementary Table 2. HCRU during follow-up among patients with SMA and reference cohorts**

|  | **Type 1 SMA** | | **Type 2 SMA** | | **Type 3 SMA** | | **UAO SMA** | |
| --- | --- | --- | --- | --- | --- | --- | --- | --- |
|  | **SMA  N = 60** | **Reference  N = 240** | **SMA  N = 55** | **Reference  N = 220** | **SMA  N = 102** | **Reference  N = 408** | **SMA  N = 73** | **Reference  N = 292** |
| **Outpatient visits (all specialties)** | | | | | | | | |
| Number of patients with ≥1 event | 53 (88.3%) | 103 (42.9%) | 55 (100.0%) | 174 (79.1%) | 102 (100.0%) | 330 (80.9%) | 72 (98.6%) | 245 (83.9%) |
| N of visits | 1,475 | 516 | 2,836 | 1,865 | 2,763 | 3,602 | 2,048 | 3,546 |
| Person-years | 143 | 572 | 488 | 1,951 | 957 | 3,729 | 725 | 2,770 |
| Annual risk (95% CI) | 0.83  (0.74–0.92) | 0.30  (0.26–0.35) | 0.94  (0.91–0.98) | 0.34  (0.30–0.37) | 0.78  (0.74–0.82) | 0.34  (0.32–0.37) | 0.77  (0.72–0.82) | 0.40  (0.37–0.43) |
| Unadjusted risk ratio (95% CI) | **2.72**  **(2.27, 3.28)** | | **2.78**  **(2.49, 3.12)** | | **2.27**  **(2.06, 2.50)** | | **1.92**  **(1.73, 2.15)** | |
| Unadjusted rate per year (95% CI) | 18.55 (13.32–23.78) | 1.33 (1.05–1.61) | 7.80 (5.52–10.07) | 1.01 (0.84–1.17) | NE | NE | 3.22 (2.42–4.01) | 1.38 (1.20–1.57) |
| Rate difference (95% CI) | **17.22 (11.99–22.45)** | | **6.79 (4.51–9.07)** | | NE | | **1.83 (1.01–2.65)** | |
| **Inpatient day cases** | | | | | | | | |
| Number of patients with ≥1 events | 10 (16.7%) | 5 (2.1%) | 26 (47.3%) | 11 (5.0%) | 18 (17.7%) | 36 (8.8%) | 8 (11.0%) | 27 (9.3%) |
| N of inpatient days | 14 | 5 | 35 | 12 | 38 | 47 | 10 | 36 |
| Person-years | 143 | 572 | 488 | 1,951 | 957 | 3,729 | 725 | 2,770 |
| Annual risk (95% CI) | 0.07  (0.02**–**0.11) | 0.01  (0.00**–**0.01) | 0.06  (0.04**–**0.08) | 0.01  (0.00**–**0.01) | 0.03  (0.01**–**0.04) | 0.01  (0.01**–**0.01) | 0.01  (0.00**–**0.02) | 0.01  (0.01**–**0.02) |
| Unadjusted risk ratio (95% CI) | **9.80**  **(3.35, 28.72)** | | **10.44**  **(5.33, 20.46)** | | **2.52**  **(1.42, 4.46)** | | 1.26  (0.56, 2.84) | |
| Unadjusted rate per year (95% CI) | 0.20 (0.04**–**0.35) | 0.01 (0.00**–**0.02) | NE | NE | 0.12 (0.01**–**0.23) | 0.01 (0.00**–**0.02) | 0.02 (0.00**–**0.05) | 0.02 (0.00**–**0.04) |
| Rate difference (95% CI) | 0.19 (0.03**–**0.35) | | NE | | 0.11 (0.00**–**0.22) | | 0.00 (0.00**–**0.03) | |
| **Inpatient overnight stays** | | | | | | | | |
| Number of patients with ≥1 events | 56 (93.3%) | 14 (5.8%) | 51 (92.7%) | 38 (17.3%) | 68 (66.7%) | 128 (31.4%) | 52 (71.2%) | 137 (46.9%) |
| N of overnight stays | 408 | 21 | 662 | 75 | 314 | 323 | 170 | 428 |
| Person-years | 143 | 572 | 488 | 1,951 | 957 | 3,729 | 725 | 2,770 |
| Annual risk (95% CI) | 0.82  (0.72**–**0.92) | 0.03  (0.01**–**0.04) | 0.66  (0.59**–**0.73) | 0.03  (0.02**–**0.04) | 0.22  (0.17**–**0.26) | 0.06  (0.05**–**0.07) | 0.15  (0.12**–**0.19) | 0.10  (0.08**–**0.11) |
| Unadjusted risk ratio (95% CI) | **29.23**  **(15.98, 53.46)** | | **23.29**  **(16.37, 33.13)** | | **3.83**  **(2.91, 5.06)** | | **1.59**  **(1.20, 2.10)** | |
| Unadjusted rate per year (95% CI) | 7.14 (5.22**–**9.05) | 0.08 (0.03**–**0.12) | 1.44 (1.14**–**1.75) | 0.08 (0.05**–**0.11) | 0.37 (0.25**–**0.48) | 0.11 (0.08**–**0.14) | 0.29 (0.18**–**0.40) | 0.22 (0.16**–**0.27) |
| Rate difference (95% CI) | **7.06 (5.14–8.98)** | | **1.37 (1.06–1.67)** | | 0.26 (0.14**–**0.38) | | 0.07 (0.00**–**0.20) | |
| **Length of stay (inpatients that have at least one overnight stay)** | | | | | | | | |
| Number of patients with ≥1 events | 56 (93%) | 14 (6%) | 51 (93%) | 39 (18%) | 70 (69%) | 133 (33%) | 53 (73%) | 140 (48%) |
| Unadjusted annual number of bed days (95% CI) | 121.14  (79.82**–**162.46) | 3.55  (0.18**–**6.93) | 6.21  (4.40**–**8.02) | 0.69  (0.45**–**0.92) | 2.80  (1.94**–**3.66) | 1.50  (1.16**–**1.84) | 3.52  (2.11**–**4.93) | 3.20  (2.40**–**3.99) |
| Difference in number of bed days (95% CI)^2^ | **117.6**  **(76.14–159.04)** | | **5.5**  **(3.69–7.35)** | | 1.3  (0.38**–**2.23) | | 0.3  (0.00**–**1.94) | |
| **Healthcare received in the municipality** | | | | | | | | |
| Number of patients with ≥1 events | 4/58 (6.9%) | 0/232 (0.0%) | 12/55 (21.8%) | 2/220 (0.9%) | 27/102 (26.5%) | 16/407 (3.9%) | 30/72 (41.7%) | 26/288 (9.0%) |
| N of events | 5 | 0 | 304 | 10 | 755 | 159 | 995 | 396 |
| Annual risk (95% CI) | NE | NE | 0.08  (0.03**–**0.13) | 0.00  (0.00**–**0.00) | 0.12  (0.07**–**0.17) | 0.01  (0.00**–**0.02) | 0.23  (0.15**–**0.31) | 0.04  (0.02**–**0.05) |
| Unadjusted risk ratio (95% CI) | NE | | **56.08**  **(11.49, 273.7)** | | **11.39**  **(5.68, 22.81)** | | **6.39**  **(3.65, 11.18)** | |
| Unadjusted rate per year (95% CI) | NE | NE | 0.60 (0.00**–**1.25) | 0.02 (0.00**–**0.05) | 0.75 (0.21**–**1.28) | 0.29 (0.02**–**0.57) | 3.17 (1.71**–**4.63) | 1.33 (0.64**–**2.03) |
| Rate difference (95% CI) | NE | | 0.58 (0.00**–**1.23) | | 0.45 (0.00**–**1.05) | | 1.84 (0.22**–**3.45) | |
| **Diagnostic and medical procedures** | | | | | | | | |
| Number of patients with ≥1 events | 54 (90.0%) | 49 (20.4%) | 55 (100.0%) | 106 (48.2%) | 93 (91.2%) | 207 (50.7%) | 69 (94.5%) | 183 (62.6%) |
| N of diagnostic and medical procedures | 1,516 | 197 | 2,666 | 1,119 | 1,940 | 2,174 | 1,233 | 2,302 |
| Person-years | 143 | 572 | 488 | 1,951 | 957 | 3,729 | 725 | 2,770 |
| Annual risk (95% CI) | 0.81  (0.71**–**0.91) | 0.10  (0.07**–**0.13) | 0.82  (0.76**–**0.88) | 0.13  (0.10**–**0.15) | 0.47  (0.42**–**0.53) | 0.14  (0.12**–**0.16) | 0.46  (0.40**–**0.52) | 0.19  (0.16**–**0.21) |
| Unadjusted risk ratio (95% CI) | **7.88**  **(5.83, 10.64)** | | **6.33**  **(5.12, 7.82)** | | **3.30**  **(2.76, 3.95)** | | **2.48**  **(2.07, 2.98)** | |
| Unadjusted rate per year (95% CI) | 32.25 (18.45**–**46.06) | 0.51 (0.33**–**0.69) | 8.00 (4.66**–**11.33) | 0.71 (0.51**–**0.91) | 2.41 (1.64**–**3.19) | 0.71 (0.56**–**0.86) | 1.99 (1.32**–**2.66) | 1.01 (0.81**–**1.22) |
| Rate difference (95% CI) | **31.74 (17.93–45.55)** | | **7.29 (3.94–10.63)** | | 1.70 (0.92**–**2.49) | | 0.98 (0.27**–**1.68) | |
| **Surgical procedures** | | | | | | | | |
| Number of patients with ≥1 events | 32 (53.3%) | 22 (9.2%) | 51 (92.7%) | 77 (35.0%) | 76 (74.5%) | 188 (46.1%) | 57 (78.1%) | 174 (60.0%) |
| N of surgical procedures | 233 | 56 | 414 | 203 | 528 | 717 | 346 | 923 |
| Person-years | 143 | 572 | 488 | 1,951 | 957 | 3,729 | 725 | 2,770 |
| Annual risk (95% CI) | 0.39  (0.29**–**0.50) | 0.04  (0.02**–**0.05) | 0.35  (0.29**–**0.41) | 0.06  (0.05**–**0.08) | 0.21  (0.18**–**0.25) | 0.10  (0.09**–**0.11) | 0.22  (0.17**–**0.27) | 0.15  (0.13**–**0.17) |
| Unadjusted risk ratio (95% CI) | **10.39**  **(6.33, 17.06)** | | **5.68**  **(4.27, 7.57)** | | **2.16**  **(1.73, 2.71)** | | **1.45**  **(1.12, 1.87)** | |
| Unadjusted rate per year (95% CI) | 2.63 (1.51**–**3.75) | 0.11 (0.06**–**0.16) | 1.21 (0.81**–**1.60) | 0.12 (0.08**–**0.15) | 0.74 (0.51**–**0.97) | 0.21 (0.16**–**0.25) | 0.52 (0.36**–**0.69) | 0.40 (0.32**–**0.48) |
| Rate difference (95% CI) | **2.52 (1.40–3.64)** | | 1.09 (0.69**–**1.49) | | 0.53 (0.30**–**0.77) | | 0.12 (0.00**–**0.30) | |
| **Prescribed medication dispensed in community pharmacies** | | | | | | | | |
| Number of patients with ≥1 events | 45 (75.0%) | 102 (42.5%) | 55 (100.0%) | 198 (90.0%) | 98 (96.1%) | 368 (90.2%) | 71 (97.3%) | 285 (97.6%) |
| N of dispensations | 3163 | 1,372 | 6,250 | 4,487 | 13,147 | 20,068 | 10,573 | 35,056 |
| Person-years | 143 | 572 | 488 | 1,951 | 957 | 3,729 | 725 | 2,770 |
| Annual risk (95% CI) | 0.78  (0.69**–**0.86) | 0.37  (0.32**–**0.42) | 0.89  (0.84**–**0.93) | 0.47  (0.43**–**0.51) | 0.71  (0.65**–**0.77) | 0.56  (0.53**–**0.59) | 0.86  (0.80**–**0.91) | 0.73  (0.70**–**0.77) |
| Unadjusted risk ratio (95% CI) | **2.07**  **(1.74, 2.47)** | | **1.90**  **(1.71, 2.10)** | | **1.27**  **(1.15, 1.41)** | | **1.17**  **(1.08, 1.27)** | |
| Unadjusted rate per year (95% CI) | NE | NE | 12.68 (8.64**–**16.71) | 2.22 (1.86**–**2.58) | 12.24 (8.68**–**15.80) | 5.74 (4.90**–**6.58) | 15.52 (10.80**–**20.25) | 14.57 (12.35**–**16.80) |
| Rate difference (95% CI) | NE | | **10.46 (6.41–14.51)** | | **6.50 (2.84–10.16)** | | 0.95 (0.00**–**6.17) | |

Abbreviations: CI = confidence interval; HCRU = healthcare resource utilisation; NE = not estimable; SMA = spinal muscular atrophy; UAO = unclassified adult onset.

Note: bold results indicate where there is a trend shown, based on CIs that do not overlap 1.

**Supplementary Table 3. Use of SMA-related medications in all patients during follow-up**

|  | **Type 1 SMA  N = 60** | **Type 2 SMA  N = 55** | **Type 3 SMA  N = 102** | **UAO SMA N = 73** |
| --- | --- | --- | --- | --- |
| **Disease-modifying therapies** | | | | |
| Nusinersen | 9/26 (35%) | 23/49 (47%) | 24/97 (25%) | 0 (0.0%) |
| **Supportive care** | | | | |
| Drugs for constipation | 22 (36.67%) | 35 (63.64%) | 33 (32.35%) | 22 (30.14%) |
| Antibacterials for systemic use | 24 (40.00%) | 49 (89.09%) | 59 (57.84%) | 49 (67.12%) |
| Antihistamines for systemic use | n < 5 | 9 (16.36%) | 18 (17.65%) | 15 (20.55%) |
| Corticosteroids for systemic use | 0 (0.00%) | n < 5 | n < 5 | 11 (15.07%) |
| Opioids | n < 5 | 15 (27.27%) | 34 (33.33%) | 31 (42.47%) |
| Other analgaesics and antipyretics | n < 5 | 20 (36.36%) | 32 (31.37%) | 27 (36.99%) |
| Drugs for obstructive airway disease | 22 (36.67%) | 46 (83.64%) | 32 (31.37%) | 14 (19.18%) |
| Proton pump inhibitors | 12 (20.00%) | 9 (16.36%) | 12 (11.76%) | 21 (28.77%) |
| Mucolytics | 5 (8.33%) | 19 (34.55%) | 18 (17.65%) | 17 (23.29%) |
| **Drugs against depression and anxiety** | | | | |
| Antidepressants | n < 5 | n < 5 | 25 (24.51%) | 23 (31.51%) |
| Anxiolytic drugs | 0 (0.00%) | n < 5 | 14 (13.73%) | 15 (20.55%) |

Abbreviations: SMA = spinal muscular atrophy; UAO = unclassified adult onset.

Note: The denominator for nusinersen is 26 for type 1, 49 for type 2, 97 for type 3, and 0 for UAO because the first intrathecal code had to occur <18-years of age i.e., when they were eligible for nusinersen in Sweden at the time of data analysis.
